# Facial Pain Mimicking Trigeminal Neuralgia Secondary to Low-Cervical Spondylosis: A Case Report and Comprehensive Literature Review

**DOI:** 10.7759/cureus.96445

**Published:** 2025-11-09

**Authors:** Gerardo Gomez-Castro, Luis A Castillejo-Adalid, Gonzalo Cancino-Gonzalez, Mario A Taylor-Martinez, Ulises Garcia-Gonzalez

**Affiliations:** 1 Department of Medicine and Nutrition, Universidad de Guanajuato, Leon, MEX; 2 Department of Neurosurgery, American British Cowdray Medical Center Private Assistance Institution (IAP), Mexico City, MEX; 3 Department of Spine Surgery, Centro Médico Nacional, Institute for Social Security and Services for State Workers (ISSSTE), Mexico City, MEX

**Keywords:** anterior cervical decompression and fusion, degenerative cervical spondylosis, facial pain, secondary trigeminal neuralgia, trigeminocervical complex

## Abstract

Trigeminal neuralgia (TN) is usually associated with neurovascular compression at the root entry zone. However, atypical presentations may result from secondary underlying pathologies. Cervical spondylosis (CS) is a rare cause, and its pathophysiologic mechanism leading to facial pain remains poorly understood. Here, we present a case of a 53-year-old woman with a one-year history of migraine episodes refractory to multiple medications. She subsequently developed sudden, severe left-sided trigeminal-like facial pain. Brain magnetic resonance imaging (MRI) excluded vascular compression or intracranial pathology, while cervical spine MRI revealed disc bulging at lower cervical levels. The patient underwent anterior cervical discectomy and fusion (ACDF) at C5-C6, achieving immediate and sustained resolution of facial pain and migraines. This case highlights the importance of evaluating the entire trigeminal pathway, including the cervical spine, when assessing patients with atypical or refractory TN, as an appropriate diagnostic approach and surgical management can lead to definitive symptom relief.

## Introduction

Trigeminal neuralgia (TN) is a characteristic and disabling facial pain condition often referred for neurosurgical treatment after failed conservative therapy [1]. The International Classification of Headache Disorders (ICHD-3) classifies TN into the following three main categories: classic, secondary, and idiopathic. Classic TN is usually caused by neurovascular compression of the trigeminal nerve root, whereas secondary TN has been associated with underlying pathologies, such as multiple sclerosis, space-occupying lesions, skull base deformities, and connective tissue diseases [2]. Although uncommon, cervical spondylosis (CS) has been associated with TN-like symptoms. A cross-sectional study of a US network database reported a 12-fold increased risk of degenerative cervical myelopathy in patients with TN [3]. However, the causality of this association remains unclear. Previous case reports [4-7], and two case series have highlighted the association of CS, most often above the C4-C5 level, with secondary TN [8,9]. Additionally, it has been hypothesized that irritation of a convergence of nerve fibers interconnecting the trigeminal nerve with head and neck structures, also known as the trigeminocervical complex (TCC), may elicit headache, facial pain, and other symptoms affecting the head and neck [10].

Here, we present an unusual case of TN-like facial pain secondary to low-CS in a woman who presented with progressive migraine episodes and recent-onset excruciating left-sided facial pain. Neurovascular compression was ruled out, and cervical imaging demonstrated a central disc bulge at C5-C6 with degenerative CS. Therefore, we aimed to outline the anatomical basis that supports this rare clinical presentation, emphasizing a careful evaluation of the entire trigeminal pathway in atypical cases of TN.

## Case presentation

Patient history

A 53-year-old woman presented with a one-year history of daily, incapacitating headaches accompanied by bilateral occipital numbness. She had previously been treated with multiple over-the-counter analgesics, two intramuscular injections of 155 units of onabotulinumtoxinA administered 12 weeks apart, and, more recently, with an initial 240 mg subcutaneous loading dose of galcanezumab followed by monthly maintenance doses of 120 mg for migraine control at another institution, without success. After the initial consultation at our institution, the patient developed continuous left hemifacial pain rated 6/10 on the numeric analog scale, exacerbated by facial touch and neck flexion and extension, prompting an emergency department visit after three days of facial pain onset. Neurologic examination revealed a limited range of motion in cervical flexion, extension, and lateral rotation. Muscle strength, sensation, deep tendon reflexes, and corneal reflexes were preserved.

Neuroimaging and neurophysiological studies

**Figure 1:**
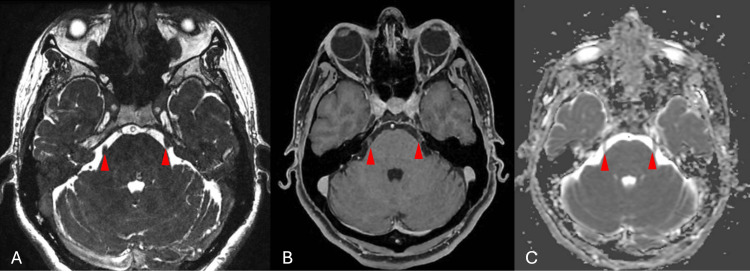
Preoperative brain MRI. (A) Axial T2-weighted FIESTA image showing no vascular compression of the root entry of the trigeminal nerves (arrowheads). (B) Axial contrast-enhanced T1 image without evidence of enhancing lesion. (C) ADC map demonstrating no restricted diffusion. The arrowheads indicate the trigeminal nerves bilaterally at the cisternal segment, demonstrating the absence of neurovascular compression. FIESTA: Fast Imaging Employing Steady-state Acquisition; ADC: apparent diffusion coefficient

**Figure 2:**
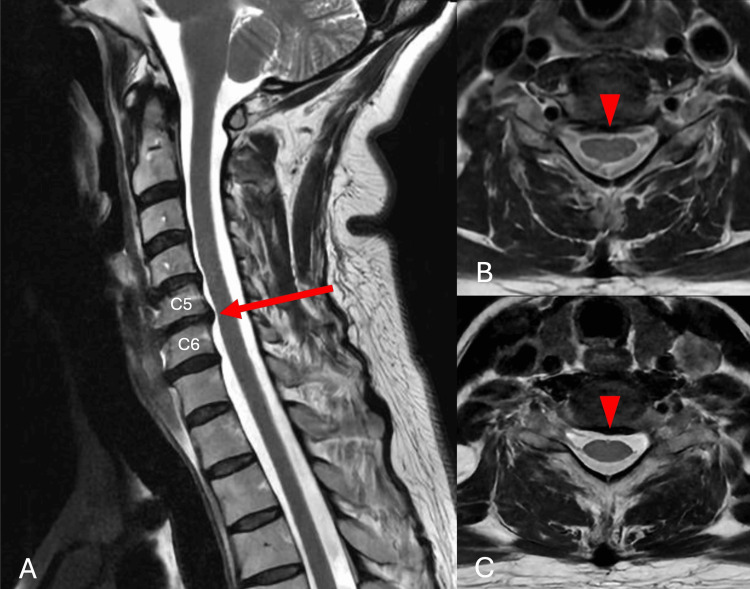
Preoperative cervical spine T2-weighted MRI. (A) Sagittal image showing disc bulging at C5-C6 (red arrow) and C6-C7 with an inferior C5 osteophyte. Axial images at (B) C5-C6 and (C) C6-C7 demonstrating central disc bulging (arrowheads) and without cord compression or foraminal stenosis.

Brain magnetic resonance imaging (MRI) showed no evidence of neurovascular compression of the trigeminal nerve (Figures 1A-1C). Subsequently, cervical spine MRI evidenced degenerative CS with C5-C6 and C6-C7 disc bulging and a large osteophytic complex indenting the anterior and posterior inferior surface of C5, causing a Kang 1 cervical canal stenosis without medullary contact or foraminal stenosis (Figures 2A-2C). Preoperative electromyography indicated C6 irritation. No trial of carbamazepine or other antiepileptic drugs was undertaken, as imaging and neurophysiologic findings supported a cervical etiology rather than primary TN.

Surgical approach and postoperative course

**Figure 3:**
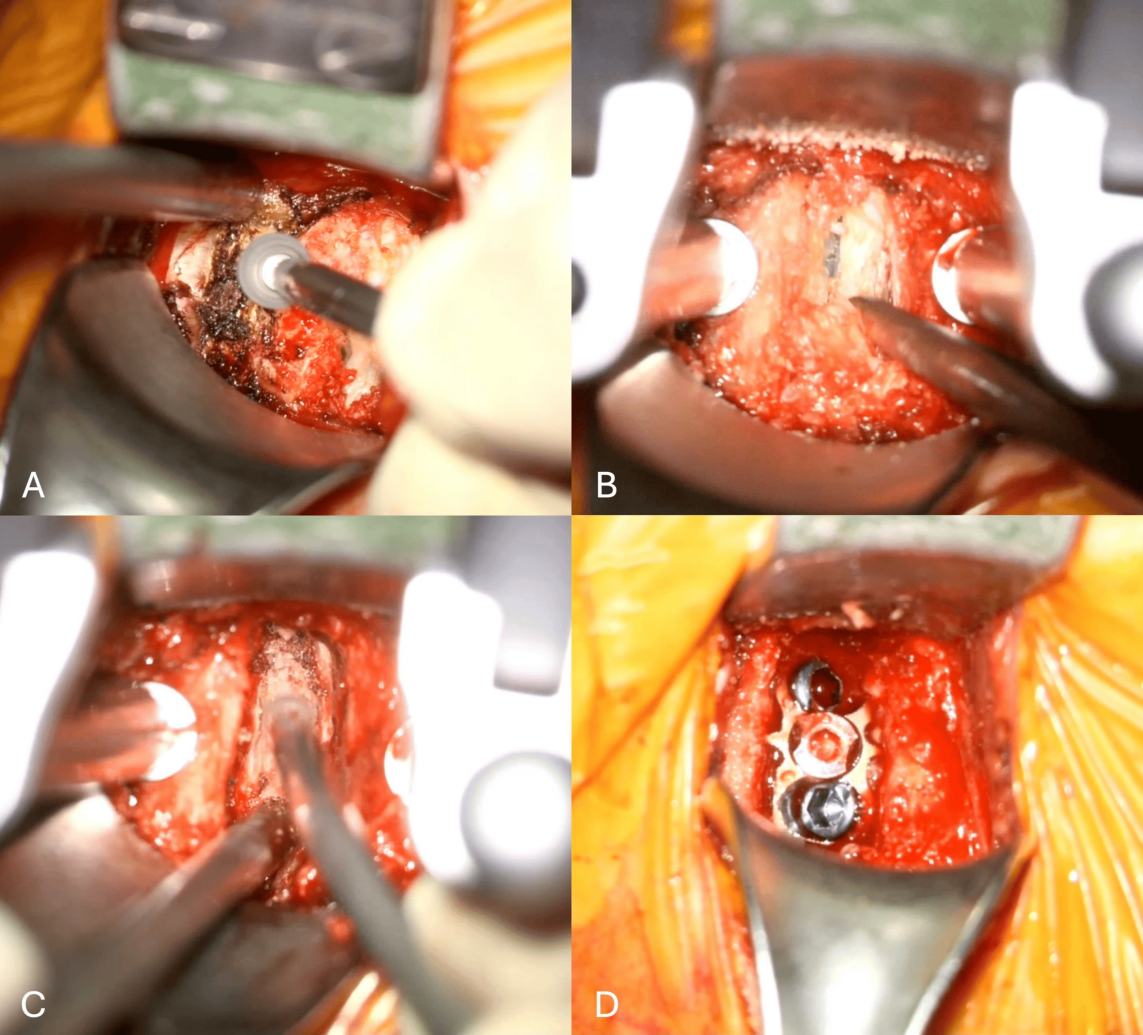
Intraoperative findings during C5-C6 anterior cervical discectomy and fusion (ACDF). (A) The anterior-inferior C5 osteophyte was removed, followed by (B) C5-C6 discectomy, (C) drilling of the posterior-inferior C5 osteophyte, and (D) stand-alone cage placement.

**Figure 4:**
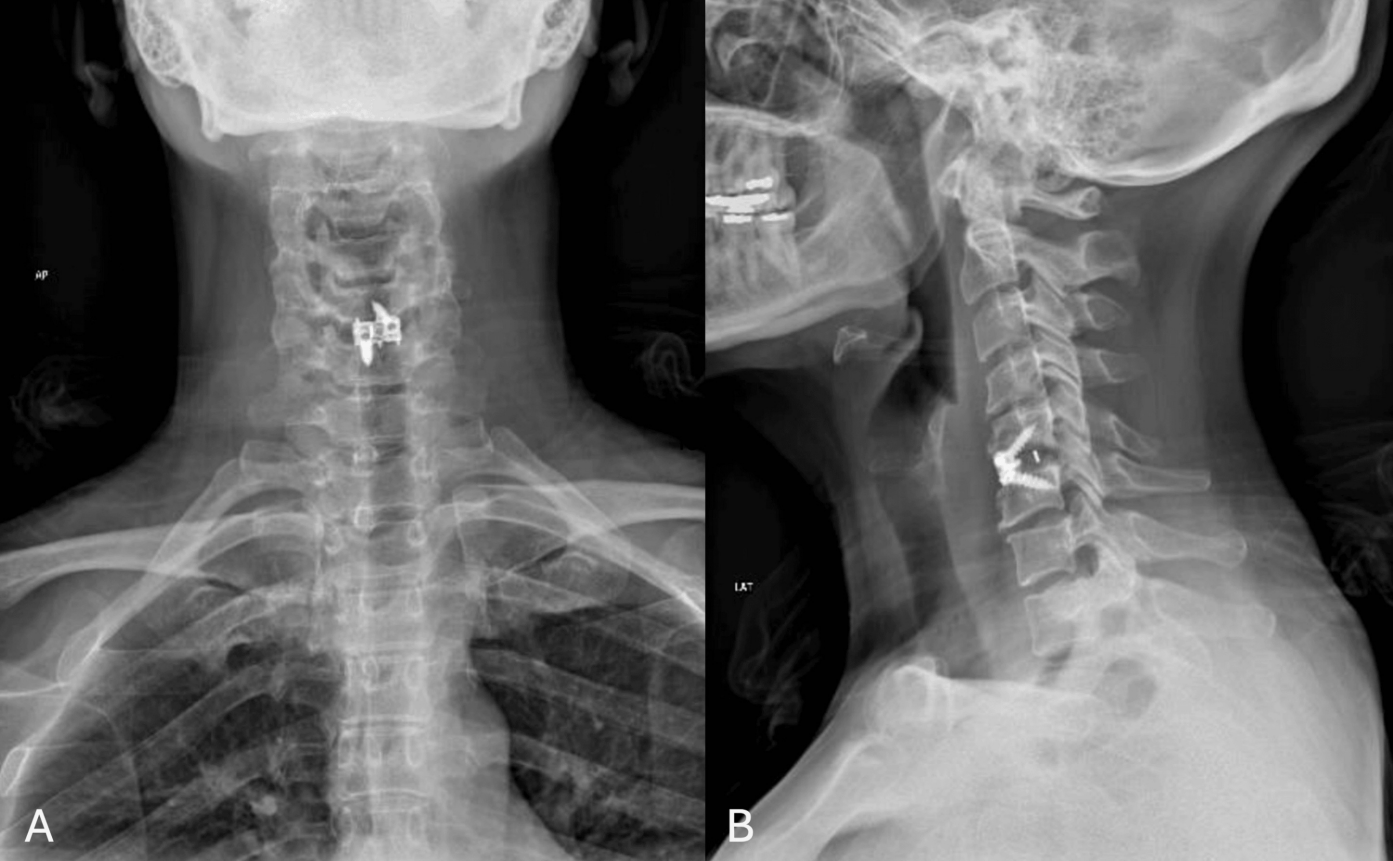
Postoperative cervical radiographs. (A) Anteroposterior view showing appropriate placement of a C5-C6 stand-alone cage. (B) Lateral view demonstrating correct cage positioning with maintained overall cervical alignment, although mild straightening of the cervical lordosis is noted.

After identifying isolated C6 irritation, we performed a C5-C6 anterior cervical discectomy and anterior cervical discectomy and fusion (ACDF) and removed the anterior and posterior osteophytic complexes at C5 (Figures 3A-3D). Upon completion of the surgical intervention, postprocedural imaging demonstrated adequate placement of the C5-C6 stand-alone cage and screw trajectory for cage fixation (Figures 4A, 4B). Postoperatively, the patient experienced complete resolution of migraine episodes, left-sided facial pain, and bilateral occipital numbness. At the six-month follow-up, the patient remained asymptomatic.

## Discussion

The initial links between cervical pathology and craniofacial pain date back to the mid-19th century, when Riadore proposed that cervical disorders could produce pain radiating from the occiput to the forehead [11], a concept later echoed by Brain as "spondylotic headaches" [12,13]. The anatomical basis for this overlap lies in the convergence of upper cervical and cranial trigeminal sensory pathways in the trigeminocervical complex (TCC), which extends from the midbrain to the upper spinal cord, reaching as low as C4, allowing bidirectional referral of painful stimuli between the neck and face [10,14]. Piovesan et al. further supported this concept by reviewing clinical and experimental evidence of trigeminocervical convergence, explaining how nociceptive signals may be referred between cervical and facial regions [15].

This mechanism gains clinical relevance given reports of TN secondary to space-occupying lesions compressing the TCC, such as cervical intramedullary tumors and vascular malformations [16,17]. In addition, Barbagli et al. reviewed 30 cases of TN secondary to CS, where 72% of cases involved levels above C4-C5, emphasizing the predominance of high-cervical involvement in such cases [7]. A summary of reported cases of TN secondary to CS is provided in Table 1.

**Table 1:** Literature review of studies reporting TN secondary to cervical spondylosis pathology. ACDF: anterior cervical discectomy and fusion; F: female; M: male; N/S: not specified; TN: trigeminal neuralgia; ROM: range of motion

Studies	Year	Number of patients	Age (years)	Sex	Level(s)	Trigeminal symptoms	Cervical spondylopathy symptoms	Approach
Barakos et al. [4]	1990	1	33	F	C3-C4	Right-sided facial numbness, loss of sensation, temperature, and touch	Neck pain, right arm, and leg paresthesia	N/S
Kuraishi et al. [5]	2016	1	49	M	C2-C4	Onion-skin hemifacial dysesthesia	Right arm paresthesia	ACDF
Francois et al. [9]	2019	3	53	F	C6	Left-sided V3 pain distribution	Daniels left rotator cuff 4/5, left brachioradialis reflex +/++++, limited cervical ROM	ACDF
			68	F	C6, C7	Facial paresthesias	Left radiculopathy C6, C7 distribution, Daniels left triceps and wrist extensors 4/5, paresthesias	
			39	F	C5, C6, C7	Tongue paresthesias	Left arm weakness and paresthesias C7 distribution, Daniels 3-4/5 in triceps and wrist flexors, Hoffman (+)	
May et al. [6]	2021	1	54	M	C4	Left-sided burning, tingling facial pain with radiation to the neck and shoulder	Mild left hemiparesis	C4 vertebrectomy and C3-C5 fusion
Börü et al. [8]	2021	20	64±12.6	N/S	C2 (1)	Trigeminal branches affected: V1 + V2 (7), V1 (6), V2 (5), V3 (2)	N/S	N/S
					C3 (5)			
					C4 (6)			
					C5 (4)			
					C6 (1)			
Barbagli et al. [7]	2025	1	49	M	C3-C4	Headaches, occipital numbness, paresthesia in palate and tongue	Limited right shoulder mobility	Posterior

In contrast, Francois et al. reported a series of three patients with lower cervical disc herniations whose trigeminal symptoms improved following ACDF [9]. This atypical presentation may be explained by the fact that although the trigeminal nucleus caudalis extends caudally to C3-C4, afferent fibers in the dorsolateral tract can ascend or descend up to three spinal cord segments before entering the dorsal horn. This theory supports the possibility that stimuli from lower cervical segments may contribute to the development of TN-like symptoms through bidirectional convergence. Notably, our patient’s low cervical lesion, continuous hemifacial pain pattern, and sustained postoperative remission further distinguish this case from those previously reported, expanding the clinical spectrum of cervicogenic trigeminal-like pain. While our findings highlight the potential role of the TCC in atypical TN, further studies incorporating intraoperative neurophysiologic mapping of trigeminal pathways are warranted to validate these mechanisms and optimize diagnostic strategies.

## Conclusions

This case highlights the importance of evaluating the entire trigeminal pathway, including the cervical spine, in patients with atypical or refractory trigeminal neuralgia. Recognizing this rare association can help prevent misdiagnosis and avoid unnecessary interventions. Although uncommon, low-cervical spondylosis may mimic trigeminal neuralgia by irritating the spinal trigeminal nuclei via the trigeminocervical complex. When imaging and neurophysiologic studies support a cervical etiology related to spondylosis, ACDF can provide definitive symptom relief when conservative management fails. Future studies incorporating trigeminal and cervical neurophysiologic monitoring or larger case series are warranted to further delineate the role of lower cervical spine in craniofacial pain syndromes.
